# Exposure to Metal Mixtures and Metabolic Syndrome in Residents Living near an Abandoned Lead–Zinc Mine: A Cross-Sectional Study

**DOI:** 10.3390/toxics13070565

**Published:** 2025-07-03

**Authors:** Min Zhao, Qi Xu, Lingqiao Qin, Tufeng He, Yifan Zhang, Runlin Chen, Lijun Tao, Ting Chen, Qiuan Zhong

**Affiliations:** 1 Department of Pharmacy, The First Affiliated Hospital of Guangxi Medical University, Nanning 530021, China; 2 Department of Epidemiology, School of Public Health, Guangxi Medical University, Nanning 530021, China; 3 School of Public Health and Health Management, Gannan Medical University, Ganzhou 341000, China; 4 Guangxi Colleges and Universities Key Laboratory of Prevention and Control of Highly Prevalent Diseases, Guangxi Medical University, Nanning 530021, China

**Keywords:** polymetallic exposure, metabolic disorders, mine tailings, epidemiological study

## Abstract

Information regarding the impact of polymetallic exposure on metabolic syndrome (MetS) among residents living near abandoned Pb-Zn mines is limited. Our objective was to investigate the impact of co-exposure to metal mixtures on the prevalence of MetS among residents. ICP-MS was used to measure the levels of 24 metals in the urine of 1744 participants, including 723 participants living near abandoned Pb-Zn mines, labeled as exposed area, and 1021 participants from other towns, labeled as reference area in the same city. Multivariable generalized linear regression, adaptive LASSO penalized regression, and BKMR were used to assess the associations between metals and MetS. The levels of eleven metals were higher, while those of nine metals were lower in the exposed area than those in the reference area. Mg, Cd, Ti, TI, Zn, Rb, and Pb were selected as important MetS predictors using LASSO regression. In exposed area, urinary Zn and TI were positively associated with MetS, whereas Mg was negatively associated with MetS. In the reference area, urinary Zn was positively associated with MetS, whereas Mg and Ti were negatively associated with MetS. The BKMR model indicates a statistically significant positive overall effect of the seven metal mixtures on MetS in the exposed area. Polymetallic exposure was positively associated with MetS risk in the abandoned Pb-Zn mining areas, suggesting that excessive Zn and TI may be associated with a higher MetS risk among residents living near abandoned Pb-Zn mines.

## 1. Introduction

Health problems associated with metal exposure are under global research attention. Humans are exposed to metals through food and various industrial processes, including mining, smelting, and battery production [1]. Mining activities can release metals into the environment long after operations have ended. Abandoned mines often leave abundant tailings, which can be a long-term source of metal contamination. In many countries, the metal pollutant concentration in the air, water, soil, and agricultural lands near abandoned mining sites is considerably greater than that in unaffected regions [2,3,4,5,6]. As a result, populations living near abandoned metal mines are potentially at greater risk of environmental pollution and health effects [7]. Thus, there is an urgent need to address the health concerns of people residing near abandoned mines.

Metabolic syndrome (MetS) is a complex disorder involving multiple interconnected components that elevate the likelihood of developing cardiovascular disease and diabetes, ultimately contributing to increased mortality [8,9]. The annual MetS prevalence has progressively increased worldwide, including in China [10,11]. Increasing evidence from studies that have been conducted in the general population suggest that some groups are at high exposure risk due to residing in highly polluted areas [12,13]. Results have shown that exposure to metals leads to MetS development [14], with elevated levels of both toxic and essential metal exposures being positively associated with MetS [15,16]. A few other studies have only detected alterations in lipid profiles, blood pressure, and glucose levels [17,18]. Variations in the exposure matrices of different metals may have resulted in inconsistent findings regarding the correlation between metal exposures and MetS. Metal exposure in population living in abandoned mine areas differs from that in general communities [19]. Lead (Pb) and zinc (Zn), two predominant metals associated with abandoned Pb-Zn mines, play important but complex roles. Pb is a non-essential toxic metal with well-known adverse health impacts, while Zn is an essential trace element that may induce toxicity at excessive levels [20]. However, there is limited information on the association between urinary metal exposure and MetS among people residing near abandoned Pb-Zn mines.

Furthermore, most previous studies have primarily focused on the impact of individual metals, whereas investigations on the combined influence of multiple metals are limited. Single-metal models have significant limitations related to high correlations among metal exposures, uncertain interactions, and linear or non-linear relationships between metals and health outcomes. Bayesian kernel machine regression (BKMR) is a novel method for evaluating the health effects of mixtures. BKMR offers versatility in modeling the combined and interactive impacts of various components. BKMR has been used to assess the effect of complex pollutant mixtures on human health and to evaluate the health effects associated with exposure to multiple combined pollutants [21,22,23].

Accordingly, this study had two primary objectives: (1) to compare urinary metal concentration profiles between residents residing near abandoned Pb-Zn mines (exposed group) and non-exposed controls, identifying significant exposure disparities; and (2) to investigate the effect of co-exposure to metal mixtures on MetS and identify metals that play key roles in multiple statistical models in the exposed population.

## 2. Materials and Methods

### 2.1. Study Population

This cross-sectional research was conducted in three towns within Liuzhou City, southwestern Guangxi, China. Environmental metal concentrations decreased progressively with increasing distance from the lead–zinc mines, with significant metal contamination observed within a 4–6 km radius of the mining area [24,25]. Therefore, inhabitants living within 5 km of the abandoned Pb-Zn mine were chosen as the exposed group, and subjects from the other two towns in the same district were chosen as the reference group of the general community. Inhabitants of the three towns had similar geographic and cultural conditions. The mine was closed in the 1980s. Between August 2015 and November 2021, we enrolled participants aged ≥ 20 years who had resided in the town for >1 year. Trained interviewers collected data on demographics, lifestyle, and medical and family history of diseases, using a standardized questionnaire. Trained health professionals, adhering to a standardized protocol, conducted anthropocentric measurements, such as body weight, height, and resting blood pressure. Blood and urine samples were taken after fasting. Ultimately, we recruited 1744 individuals who provided complete clinical data, questionnaires, and biological samples. The study protocol received approval from the Ethics Committee of Guangxi Medical University (20180312-096). All subjects provided written informed consent before participation.

### 2.2. Analysis of Urinary Metals

The concentrations of metals (Ca, Ti, V, Cr, Mn, Fe, Co, Ni, Cu, Zn, Ga, As, Se, Rb, Sr, Mo, Cd, Sn, Sb, Cs, W, Tl, and Pb) in urine were determined using an inductively coupled plasma-mass spectrometer (ICP-MS; iCAPRQ01408, Thermo Fisher Scientific, Waltham, MA, USA). High-purity argon gas (≥99.999%, Guangxi Ruida Gas, Nanning, Guangxi, China) was used as the plasma, auxiliary gas and nebulizer gas. Helium (≥99.999%, Guangxi Ruida Gas, Nanning, Guangxi, China) was used as a collision gas during measurement. Before analysis, urine samples were frozen and stored at −80 °C. Samples were gradually rewarmed. They were thawed at −20 °C for 24 h and 4 °C for 24 h, respectively. After complete thawing, 200 µL of each urine sample was diluted to 2.0 mL with 1% (*v*/*v*) nitric acid. The 1% nitric acid was prepared using ultra-pure nitric acid (HNO_3_, Suprapur^®^ grade, Merck, Darmstadt, Germany) and ultrapure water (resistivity 18.2 MΩ·cm). The samples were then acidified overnight at 4 °C. Before chemical analysis, the urine samples were digested by ultrasound at 40 °C for 30 min. Calibration curves were established for each metal using multi-element standard solutions (Inorganic Ventures, Christiansburg, VA, USA) diluted in 1% nitric acid. Internal standards (Sc, Y, Ge, Tb, In, Rh, Re, and Bi at a concentration of 50 μg/L each) were added to correct for matrix effects and instrumental drift. Four calibration curves were prepared according to the concentration ranges of different metals, each containing six concentration levels covering expected urinary concentrations. Calibration curves exhibited correlation coefficients (R^2^) ≥ 0.999 for all elements analyzed. Each sample was measured three times, and the mean value was determined. Quality control was conducted utilizing urinary standard reference materials (Seronorm^TM^ Trace Elements Urine Level-1 and Level-2, Sero AS, Billingstad, Norway) once in every 25 samples. The concentrations of each reference metal was within the recommended range. We used spike recovery tests for Ca, Ti, Ga, Rb, Sr, Mo, Cs, and W since the urinary standard reference materials did not provide quality control results for them. Spike recovery tests were conducted with low, medium, and high concentration levels of standard solutions to determine their recovery rates. Detailed results were provided in Supplementary Tables S1 and S2. The spiked recovery rates were ranged from 78.5% to 119.8%, and the inter-day and intra-day coefficients of variation were both less than 10%. The limits of detection (LOD) varied between 0.00008 and 3.70 μg/L. Urinary metal levels were creatinine adjusted, with measurements obtained using an automated clinical biochemistry analyzer (Hitachi 7600, Hoachi, Kyoto, Japan). Results were reported in μg/g creatinine.

### 2.3. Biochemical Measurements

Blood samples were sent to the First Affiliated Hospital of Guangxi Medical University through cold-chain transportation to determine blood biochemical indicators, including high-density cholesterol (HDL-c), serum triglyceride (TG), total cholesterol (TC), low-density cholesterol (LDL-c), and fasting plasma glucose (FPG). Blood biochemical indices were analyzed using an automatic biochemistry analyzer (Hitachi 7600, Hoachi, Kyoto, Japan), following the manufacturer’s guidelines.

### 2.4. Definition of MetS

Considering the guidelines established by the China Diabetes Federation, MetS was diagnosed when participants met ≥ 3 of these criteria: (1) abdominal obesity (BMI ≥ 25 kg/m^2^, both sexes); (2) dyslipidemia (TG ≥ 1.7 mmol/L or HDL-c < 0.9 mmol/L [male]/<1.0 mmol/L [women]); (3) high blood pressure (SBP ≥ 140 mmHg, or DBP ≥ 90 mmHg, or anti-hypertensive treatment); and (4) hyperglycemia (FPG ≥ 6.1 mmol/L, or hypoglycemic medications) [26].

### 2.5. Covariates

A structured and standardized questionnaire was used to gather data regarding age, ethnicity, education level, smoking habits, alcohol intake, and physical activity. Education was categorized into two groups: junior school or lower, and high school or higher. Self-reported alcohol intake was grouped into never, former, and current drinkers. Smoking status was classified into current, never smoked, and former smokers. The metabolic equivalent (MET) of physical activity was determined by multiplying the MET coefficient for specific activity by duration (minutes per week) and frequency (times per week). According to the guidelines of the International Physical Activity Questionnaire (IPAQ), physical activity was categorized into three levels: low, moderate, and high [27]. These covariates were selected, considering their known associations between urinary metal concentrations and MetS.

### 2.6. Statistical Analysis

Urinary metal concentrations below the LOD were designated as LOD/√2. The concentrations of urinary metals were adjusted based on the urinary creatinine levels. Creatinine-adjusted metal concentrations were subjected to natural log transformation to achieve a normal distribution. Continuous data were presented as means with standard deviations or medians with interquartile ranges, whereas categorical data were reported as percentages. To assess the statistical differences in demographic features and metal concentrations among participants with and without MetS, *t*-tests or the Wilcoxon rank-sum test were used for continuous data, and chi-square tests were applied for categorical data. The Spearman rank correlation coefficient was performed to assess the correlations among the metals.

We performed the adaptive least absolute shrinkage and selection operator (LASSO) penalized regression to identify the key metals linked to MetS prevalence in all participants by adjusting for age (<60 years and ≥60 years), sex (men and women), ethnicity (Han, Zhuang, and others), education (less than high school, at least high school), smoking habits (never, former, and current), alcohol consumption (never, former, and current), physical activity (low, moderate, and high), BMI (continuous), and residence area (exposed and reference). We implemented a 10-fold cross-validation to establish the optimal value of lambda.

Multilevel mixed-effects logistic regression models were used to evaluate the association between the metals selected by the LASSO regression models and the MetS risk in all participants. Individuals were set at the first level, and the residential area was set at the second level. The analyses were stratified considering the residential area. The urinary metal levels were divided into four quartiles considering the creatinine-adjusted levels. Logistic regression models were conducted to assess the adjusted odds ratios (ORs) and 95% confidence intervals (CIs) for MetS by comparing quartiles 2–4 of urinary metal concentrations to the lowest quartile. The trend across the quartiles of urinary metals was tested by treating the median value of each quartile as a continuous predictor in the regression models. We used the BKMR model to evaluate the combined effects and potential interactive effects of multiple metals on MetS. This approach was flexibly executed with 50,000 iterations using the Markov Chain Monte Carlo method alongside a Gaussian kernel function. We excluded subjects with a residence duration less than 40 years for the sensitivity analysis. We also used a quantile G-computation model to evaluate the impact of mixed exposure.

All analyses were conducted using Stata (17.0) software (StataCorp, College Station, TX, USA) and R (4.2.0) (RStudio, Boston, MA, USA), and a two-sided *p*-value of <0.05 was considered statistically significant.

## 3. Results

### 3.1. Population Characteristics

Among the 1744 participants, 723 and 1021 were from exposed and reference areas, respectively. For both areas, participants with MetS were more likely to be older, less educated, have higher BMI, DBP, SBP, TG, TC, LDL-c, and FPG levels, and lower HDL-c levels than those without MetS (Table 1).

### 3.2. Urinary Concentrations of Metals

Table 2 summarizes the urinary metal concentration profiles for both groups. Compared to the reference group (residents from non-mining areas), the exposed group (individuals living near the closed mine) exhibited significantly elevated urinary concentrations of Cr, Mn, Fe, Co, Cu, As, Mo, Cd, Sn, W, and Pb, and significantly reduced levels of Mg, Ti, V, Se, Rb, Sr, Sb, Cs, and Tl. The correlation plots for the 24 metals are illustrated in Figure S1. The range of Spearman’s rank correlation coefficients (r) among the metals varied from −0.01 to 0.78, with the majority of metals demonstrating positive correlations. The highest correlation was observed between Rb and Cs concentrations (r = 0.78).

### 3.3. Single-Metal Logistic Regression Analyses

Data from all participants (*n* = 1744) were analyzed following adjustment for age, sex, ethnicity, education attainment, smoking habits, alcohol use, physical activity, BMI, and residential area. Single-metal logistic regression models revealed that Cu and Zn statistically and positively associated with MetS, whereas Mg, Ti, V, and Sn significantly and negatively associated (Table S3). In the exposed areas, single-metal models revealed that Cu, Zn, Se, and Tl significantly associated with MetS (Table S4). The single-metal models in the reference area revealed that Mg, Ca, Ti, V, Cr, Mn, Cu, Zn, Se, Sr, Sn, and Cs significantly associated with MetS (Table S5).

### 3.4. Selecting Important Metals Associated with MetS and Multiple Metals Logistic Regression Analyses

**Figure 1:**
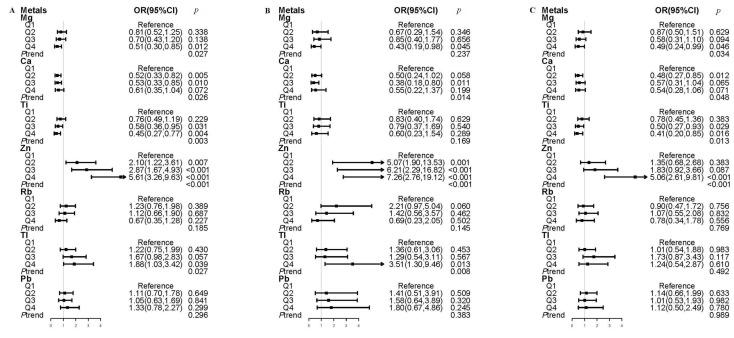
Adjusted OR (95% CI) for MetS according to quartiles of urinary metals in the multiple-metal models. (**A**) All participants, (**B**) in exposed area, (**C**) in reference area. The metals selected by LASSO analysis were simultaneously included in the logistic regression models, with adjustment for age, sex, ethnicity, levels of education, smoking habits, drinking status, physical activity, BMI, and residence area as a random effect in the all participants.

The adaptive LASSO-penalized regression models selected seven metals, including Mg, Ca, Ti, Tl, Zn, Rb, and Pb, as significant predictors of MetS, with non-zero coefficients when the minimal value of lambda (lambda = 0.003) was chosen. Multiple metal logistic regression models simultaneously incorporated these metals to assess their effects on MetS. The multiple-metal models indicated a significant positive correlation between MetS and Zn and Tl, whereas Mg, Ca, and Ti demonstrated a significant negative correlation with MetS in all participants (Figure 1A).

In the exposed area, urinary Zn and Tl were significantly and positively associated with MetS, and the corresponding ORs for MetS comparing the highest quartile to those in the lowest quartile were 7.26 (95% CI: 2.76, 19.12) and 3.51 (95% CI: 1.30, 9.46), respectively. The OR for MetS comparing the fourth quartile of urinary Mg with the first quartile was 0.43 (95% CI: 0.19, 0.98) (Figure 1B). In the reference area, a significant positive correlation was observed between Zn level and MetS. Mg and Ti were significantly negatively associated with MetS. The ORs comparing the fourth quartile to the first quartile were 5.06 (95% CI: 2.61, 9.81) for Zn, 0.49 (95% CI: 0.24, 0.99) for Mg, and 0.41 (95% CI: 0.20, 0.85) for Ti (Figure 1C). 

### 3.5. Bayesian Kernel Machine Regression Models

**Figure 2:**
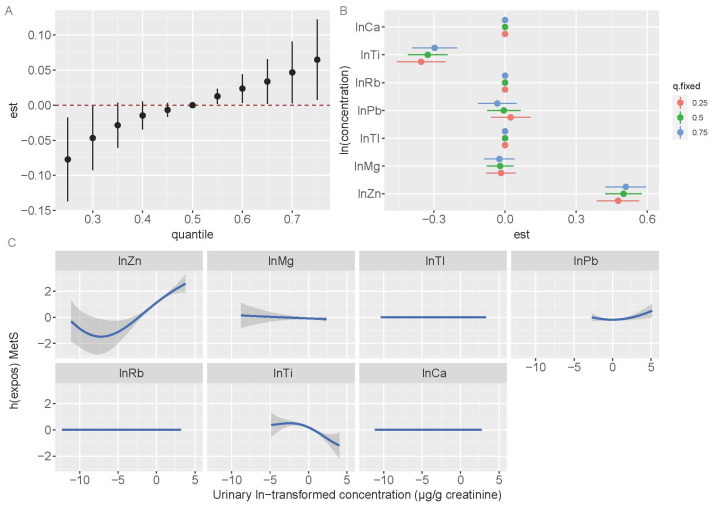
The mixture’s combined impact on metabolic syndrome in all participants. Age, sex, ethnicity, education attainment, smoking habits, drinking status, physical activity, BMI, and residence area were adjusted while estimating the data using BKMR. (**A**) Overall effect of the mixture estimates and 95% CI. The red dotted line indicates the p50 reference. (**B**) The single-exposure effect and 95% CI between urinary metals concentrations and metabolic syndrome when one metal is at the 75th percentile as compared to its 25th percentile, while the other metals are set to p25, p50, and p75. (**C**) The 95% CI (shaded regions) and univariate exposure–response function for each metal, with the concentrations of other metals held at median values.

The BKMR models revealed a joint association between the seven metals and MetS in all participants. When the seven metals were >50th percentile, the metal mixtures exhibited a considerable positive relationship with MetS compared with that when the metals were at the 50th percentile (Figure 2A). The estimated changes in MetS for a particular metal increased from the 25th to the 75th percentiles, whereas other metals were at varying percentiles (p25, p50, or p75) (Figure 2B). With the other six metals constrained to p25, p50, or p75, Zn showed a significant positive association with MetS. However, the Ti levels exhibited a negative association at p25, p50, or p75. The dose–response relationship for Zn exhibited a non-linear pattern while holding the levels of other metals constant at their median values. The exposure-response to Ti was consistent with the findings from the single-metal logistic regression models and multiple-metal logistic regression analysis (Figure 2C).

**Figure 3:**
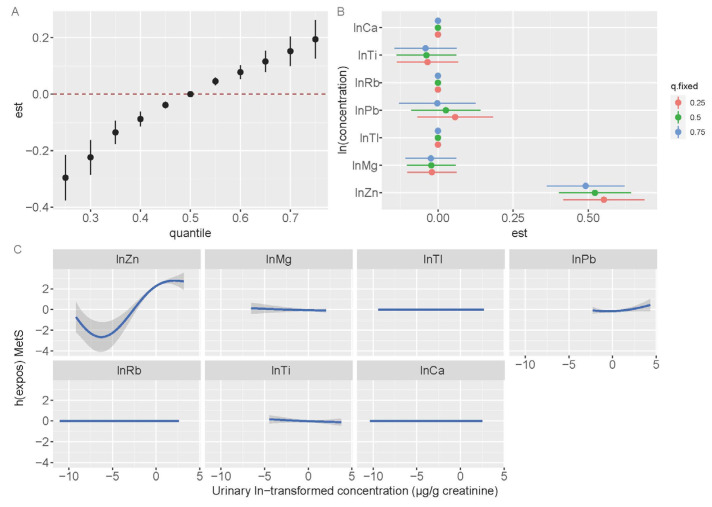
The mixture’s combined impact on metabolic syndrome in the exposed area. Age, sex, ethnicity, education attainment, smoking habits, drinking status, physical activity, and BMI were adjusted while estimating the data using BKMR. (**A**) Overall effect of the mixture estimates and 95% CI. The red dotted line indicates the p50 reference. (**B**) The single-exposure effect and 95% CI between urinary metals concentrations and metabolic syndrome when one metal is at the 75th percentile as compared to its 25th percentile, while the other metals are set to p25, p50, and p75. (**C**) The 95% CI (shaded regions) and univariate exposure-response function for each metal, with the concentrations of the other metals held at median levels.

In the exposed areas, we found that the risk of MetS increased with increasing metal concentrations. The statistical significance of the joint effect was noted when metal concentrations reached or exceeded their 25th percentile compared with that when the metals were at the 50th percentile (Figure 3A). The estimated changes in MetS for a specific metal rose from the 25th to the 75th percentile while the levels of other metals held constant at varying percentiles (p25, p50, or p75) (Figure 3B). When other metals were fixed at p25, p50, or p75, urinary Zn exhibited a significant positive association with MetS at all percentiles. Figure 3C illustrates the exposure–response relationships with other metals maintained at their median levels. A non-linear correlation was identified between Zn levels and MetS.

**Figure 4:**
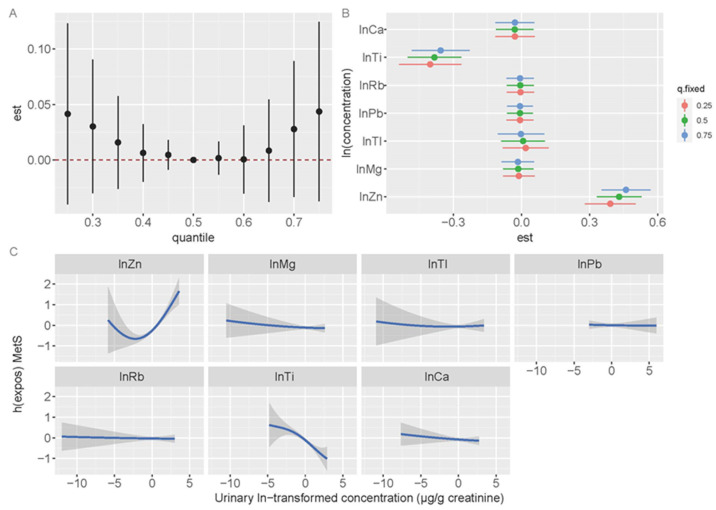
The mixture’s combined impact on metabolic syndrome in the reference area. Age, sex, ethnicity, education attainment, smoking habits, drinking status, physical activity, and BMI were adjusted while estimating the data using BKMR. (**A**) Overall effect of the mixture estimates and 95% CI. The red dotted line indicates the p50 reference. (**B**) The single-exposure effect and 95% CI between urinary metal concentrations and metabolic syndrome when one metal is at the 75th percentile as compared to its 25th percentile, while the other metals are set to p25, p50, and p75. (**C**) The 95% CI (shaded regions) and univariate exposure-response function for each metal, with the concentrations of other metals held at median values.

In the reference area, no significant association was observed between the metal mixture and MetS (Figure 4A). When other metals were set at p25, p50, or p75, urinary Zn changed from the 25th to the 75th percentile showed a significant and positive association with MetS. However, urinary Ti was significantly and negatively correlated with MetS at p25, p50, and p75 (Figure 4B). Additionally, we assessed the exposure–response relationships for individual metals while keeping the concentrations of the other metals constant at their median values (Figure 4C). Our analysis revealed a positive association between Zn and MetS, whereas Ti was negatively associated with MetS. Additionally, a non-linear relationship was identified between the Zn levels and MetS.

### 3.6. Sensitivity Analysis

After excluding 492 subjects who had resided in the study area for less than 40 years, the metal exposure maintained a significant association with MetS. In the exposure area, Zn and Tl were significantly and positively correlated with MetS (Figure S2A). Zn showed a significant positive association with MetS, while Ti demonstrated an inverse association with MetS in the reference area (Figure S2B). The quantile g-computation model revealed significant positive associations of multiple metal exposure with MetS in the exposure area (0.42, 95% CI: 0.05, 0.78; *p* = 0.026), with Zn and Tl contributing the most positive weights (Figure S3A). No significant association was observed between multiple metal exposures and MetS in the reference area (−0.12, 95% CI: −0.46, 0.22; *p* = 0.495), where Zn and Ti exhibited the largest positive and negative weights, respectively (Figure S3B).

## 4. Discussion

Our analysis revealed the association between co-exposure to metal mixtures and MetS varied across the different areas. In summary, Zn and Tl were positively correlated with MetS in the abandoned Pb–Zn mine area. In the general community, urinary Zn and Ti levels were positively and negatively associated with MetS, respectively. For all the participants, the corresponding association was consistent. Additionally, we discovered varying joint associations between metal mixtures and MetS in the two areas. The BKMR models suggested that the combined impact of multiple metals was associated with a higher MetS risk in the abandoned Pb-Zn mine area. No significant interactions were found among the different metals in the general population.

Urine metals are reliable indicators that reflect systemic exposure and internal metal burden. In this study, the median level of urinary Zn was 272.35 μg/g creatinine, exceeding the levels reported in the general Chinese population (median = 208.30 μg/g creatinine) [28], and exceeding those reported in Germany (median = 196.00 μg/g creatinine) [29]. The urinary Cd concentration (median = 2.86 μg/g creatinine) in our study was higher than that of the general Chinese population (median = 0.68 μg/g creatinine) [28], and those in Japan [30], Korea [31], and Germany [29]. Similarly, the urinary Tl concentration (median = 0.46 μg/g creatinine) in our study surpassed that of the general Chinese population (median = 0.37 μg/g creatinine) [32]. The Mg urinary concentration (median = 3.7 × 104 μg/g creatinine) was lower than that of the general Chinese population (median = 4.08 × 104 μg/g creatinine) [32]. The Cu, Pb, and As urinary concentrations were higher than those in the general population [33,34,35]. Contrastingly, the Cr, Co, and Mo concentrations were comparable to those in the general population [36,37]. We found that residents living near abandoned Pb-Zn mines had different exposure levels to different metals, with some being low, such as Mg, and some being high, such as Zn, Cd, Tl, Pb, and As. In general, human exposure to metals in the areas surrounding abandoned metal mines is higher than that of the general population, consistent with findings of previous studies. Inhabitants residing near abandoned metal mines may be exposed to multiple metals long after mining activities have stopped.

In our study, Zn emerged as a significant risk factor for MetS, aligning with prior research. A previous study have demonstrated a positive correlation between urinary Zn levels and MetS and its individual components [12]. Another previous study identified a positive correlation between serum Zn levels and increased MetS risk [15]. Conversely, several other investigations have reported negative or non-significant associations [38,39]. These inconsistent findings may be due to variations in the study population, matrices used, or criteria employed to define MetS. Although epidemiological studies have suggested that Zn influences MetS development, the underlying biological mechanisms remain unclear. Zn is an essential trace metal that regulates metabolism, particularly lipid and glycemic metabolism [40]. However, Zn is a heavy metal and a trace element. Excessive Zn can lead to the release of reactive oxygen species, resulting in severe mitochondrial dysfunction and oxidative stress, which may contribute to metabolic disorders [41,42].

Tl is a naturally occurring trace element commonly found throughout the Earth’s crust. It is regarded as one of the most toxic metals [43]. Our study identified a positive relationship between urinary Tl level and MetS. Relatively few previous studies have investigated the relationship between Tl level and MetS. A previous study reported a positive correlation between Tl and obesity [44]. Tl is a cumulative toxin associated with multiple adverse health outcomes. Although its toxicity mechanisms are not fully elucidated, emerging evidence suggests that Tl-mediated oxidative stress may disrupt metabolic homeostasis by promoting lipogenesis while suppressing energy production [44].

Our findings suggest that Mg may have a beneficial effect on MetS. Consistently, a cross-sectional study in Austria found that elevated urinary Mg levels significantly correlated with decreased MetS odds and obesity [45]. The low Mg levels in spot urine samples may have resulted from insufficient Mg intake. The National Health and Nutrition Examination Survey (NHANES) conducted in the United States noted a strong association between higher Mg intake and a significant reduction in obesity and MetS prevalence [46]. Similarly, a study conducted in a large Chinese population reported an inverse relationship between Mg dietary intake and MetS prevalence [47]. Mg is a cofactor in numerous important enzymatic reactions. As an integral part of ATP metabolism, Mg plays key roles in various metabolic processes, including glucose utilization, energy production, and the synthesis of lipids, proteins, and nucleic acids. Mg may exert anti-obesity effects by forming insoluble soaps with intestinal fatty acids, and it reduces the dietary digestible energy content [48].

Previous epidemiological studies have primarily focused on individual metals. However, the toxicity associated with metal combinations is different from that associated with individual metals. However, research on the complex effects of metal mixtures on MetS is limited. A previous study built an environmental risk score to assess the effect of metal mixtures on MetS [12] and observed a significant positive cumulative effect of the mixture on MetS. Another previous study used BKMR to examine the relationship between essential metal exposure and metabolic syndrome [13] and found that higher levels of metal mixtures were significantly associated with an increase in MetS. Consistent with these findings, our findings demonstrated a significant association between metal exposure and increased MetS risk. The results of this study have important public health implications. The study revealed that heavy metal exposure related to the risk of MetS in inhabitants living near abandoned Pb-Zn mines is increasing, and the Zn, Tl, and Mg internal exposure levels require future risk monitoring. The present study revealed that exposure to essential metals such as Zn and Mg is related to MetS, potentially offering new evidence for the management and regulation of essential metals. An optimal amount of essential metals is required for the body, and excessive or insufficient amounts can lead to adverse health effects.

We investigated the population in general communities and those living near an abandoned mine simultaneously, enhancing the specificity and robustness of the research results in abandoned mining areas. Different statistical methods were used to assess the correlations and weights of each MetS marker and 24 metals were detected, which effectively reduced the confounding effects of various metals. The study design precludes inferring causality; it catered for reverse causality, where some of the MetS risk factors or components may contribute to higher element exposure through diet, but the disease may also contribute to higher excretion of elements via urine. Nonetheless, this study had limitations. First, it employed a cross-sectional design, which restricted the ability to deduce causal interactions between urinary metals and MetS. Second, the collection of data regarding the physical activity, smoking habits, and alcohol consumption of the participants using a questionnaire may have introduced bias. Third, although several confounders were adjusted for in our analysis, other factors, such as eating habits and other chemicals, were not included despite their reported associations with MetS [49,50]. Additionally, we did not collect data on comorbid conditions such as anemia or chronic obstructive pulmonary disease (COPD), which may influence metal metabolism, excretion, and body burden [51].

## 5. Conclusions

In this study, the abandoned metal mine area exhibited different exposure profiles of urinary metals compared with those of the general community. Furthermore, we found that the MetS risk increased with increasing urinary metal mixture values, including those of Mg, Cd, Ti, Tl, Zn, Rb, and Pb, in the abandoned Pb-Zn mine area. Urinary Zn and Tl levels were positively correlated with MetS in individuals living near abandoned Pb-Zn mines. Future studies should confirm the results of this study and clarify the underlying mechanisms.

## Figures and Tables

**Table 1:** Descriptive characteristics of the 1744 subjects.

Variables	Reference Area		Exposed Area	
	MetS (*n* = 170)	No MetS (*n* = 851)	MetS (*n* = 99)	No MetS (*n* = 624)
Age, years	58.4 ± 8.7	55.4 ± 10.7	57.2 ± 10.3	54.5 ± 12.7
Sex, n				
Male	69 (40.6%)	260 (30.6%)	40 (40.4%)	231 (37.0%)
Female	101 (59.4%)	591 (69.4%)	59 (59.6%)	393 (63.0%)
Ethnicity, n				
Han	115 (67.6%)	458 (53.8%)	20 (20.2%)	126 (20.2%)
Zhuang	17 (10.0%)	197 (23.1%)	74 (74.7%)	480 (76.9%)
Others	38 (22.4%)	196 (23.0%)	5 (5.1%)	18 (2.9%)
Education, n				
<high school	158 (92.9%)	771 (90.6%)	89 (89.9%)	512 (82.1%)
≥high school	12 (7.1%)	80 (9.4%)	10 (10.1%)	112 (17.9%)
Smoking status, n				
Never	126 (74.1%)	653 (76.7%)	73 (73.7%)	474 (76.0%)
Former	26 (15.3%)	70 (8.2%)	7 (7.1%)	58 (9.3%)
Current	18 (10.6%)	128 (15.0%)	19 (19.2%)	92 (14.7%)
Drinking status, n				
Never	74 (43.5%)	385 (45.2%)	45 (45.5%)	335 (53.7%)
Former	31 (18.2%)	130 (15.3%)	14 (14.1%)	65 (10.4%)
Current	65 (38.2%)	336 (39.5%)	40 (40.4%)	224 (35.9%)
Physical activity, n				
Low	5 (2.9%)	29 (3.4%)	2 (2.0%)	25 (4.0%)
Moderate	36 (21.2%)	105 (12.3%)	28 (28.3%)	82 (13.1%)
High	129 (75.9%)	717 (84.3%)	69 (69.7%)	517 (82.9%)
BMI, kg/m^2^	27.1 ± 3.1	23.0 ± 3.2	26.4 ± 3.1	22.3 ± 3.0
DBP, mmHg	89.5 ± 12.3	78.7 ± 12.1	87.0 ± 15.2	74.9 ± 12.5
SBP, mmHg	152.7 ± 20.3	135.1 ± 20.5	154.1 ± 22.3	133.2 ± 22.2
TG, mmol/L	2.25 (1.82, 3.44)	1.34 (0.97, 1.87)	2.34 (1.75, 3.32)	1.08 (0.80, 1.49)
TC, mmol/L	5.61 (4.89, 6.52)	5.21 (4.60, 5.86)	5.85 (5.29, 6.34)	5.08 (4.51, 5.89)
HDL-c, mmol/L	1.25 (1.11, 1.42)	1.52 (1.30, 1.80)	1.30 (1.07, 1.57)	1.44 (1.24, 1.71)
LDL-c, mmol/L	3.02 (2.54, 3.84)	2.89 (2.35, 3.52)	3.03 (2.55, 3.53)	2.74 (2.26, 3.32)
FPG, mmol/L	6.42 (5.63, 7.88)	5.46 (5.16, 5.79)	6.38 (5.80, 7.14)	5.44 (5.07, 5.85)

Abbreviations: SD, standard deviation; BMI, body mass index; DBP, diastolic blood pressure; SBP, systolic blood pressure; TG, triglyceride; TC, total cholesterol; HDL-c, high-density lipoprotein cholesterol; LDL-c, low-density lipoprotein cholesterol; FPG, fasting plasma glucose; MetS, metabolic syndrome. Categorical variables are summarized as counts (percentages), whereas continuous variables are expressed as mean ± SD for normally distributed data or median (p25, p75) for non-parametrically distributed data.

**Table 2:** Comparison of urinary metal concentrations between the exposed group and the reference group.

Metals (μg/g Creatinine)	<LOD (%)	Reference (*n* = 1021)	Exposed (*n* = 723)	Total (*n* = 1744)	*p*-Value
Mg (mg/g creatinine)	0.00	38.38 (25.15, 53.35)	37.10 (17.51, 57.37)	37.68 (23.02, 55.48)	0.026
Ca (mg/g creatinine)	0.06	85.478 (51.75, 130.81)	86.07 (51.25, 138.59)	85.81 (51.53, 134.20)	0.300
Ti	0.00	22.96 (16.81, 30.82)	21.61 (15.25, 28.89)	22.50 (16.27, 30.05)	0.004
V	0.00	0.24 (0.16, 0.34)	0.21 (0.15, 0.29)	0.22 (0.16, 0.32)	<0.001
Cr	0.17	0.26 (0.16, 0.48)	0.50 (0.28, 1.25)	0.34 (0.19, 0.74)	<0.001
Mn	0.00	0.25 (0.13, 0.56)	0.44 (0.21, 0.92)	0.32 (0.15, 0.72)	<0.001
Fe	0.00	13.86 (9.24, 23.88)	15.48 (9.20, 28.56)	14.36 (9.23, 25.59)	0.036
Co	0.00	0.25 (0.16, 0.51)	0.29 (0.17, 0.63)	0.27 (0.16, 0.55)	0.013
Ni	0.34	1.62 (1.00, 2.70)	1.56 (0.91, 2.68)	1.61 (0.97, 2.69)	0.200
Cu	0.00	12.26 (9.55, 16.22)	13.50 (10.18, 19.56)	12.63 (9.77, 17.52)	<0.001
Zn	0.17	272.43 (197.58, 383.70)	272.04 (180.04, 396.00)	272.35 (188.94, 388.68)	0.464
Ga	0.57	0.24 (0.13, 0.46)	0.27 (0.14, 0.53)	0.25 (0.14, 0.48)	0.053
As	0.00	34.31 (24.53, 49.10)	37.89 (25.09, 54.42)	35.58 (24.66, 51.29)	0.004
Se	0.00	23.73 (18.83, 29.89)	18.21 (13.32, 23.74)	21.33 (16.63, 27.80)	<0.001
Rb (mg/g creatinine)	0.00	1.75 (1.28, 2.578)	1.66 (1.17, 2.24)	1.71 (1.24, 2.42)	<0.001
Sr	0.06	80.89 (52.16, 122.05)	46.48 (27.96, 72.91)	65.82 (38.64, 103.21)	<0.001
Mo	0.00	68.87 (45.75, 104.90)	91.19 (55.14, 150.85)	76.66 (48.79, 126.29)	<0.001
Cd	0.00	2.34 (1.39, 3.83)	4.15 (2.12, 8.08)	2.86 (1.60, 5.22)	<0.001
Sn	0.11	0.48 (0.34, 0.74)	0.62 (0.42, 0.93)	0.53 (0.37, 0.84)	<0.001
Sb	0.11	0.09 (0.06, 0.14)	0.07 (0.05, 0.11)	0.08 (0.06, 0.13)	<0.001
Cs	0.00	8.85 (6.71, 11.87)	7.58 (5.59, 9.77)	8.21 (6.21, 10.85)	<0.001
W	0.52	0.23 (0.11, 0.59)	0.39 (0.15, 1.25)	0.28 (0.12, 0.85)	<0.001
Tl	0.00	0.47 (0.33, 0.70)	0.45 (0.29, 0.67)	0.46 (0.31, 0.69)	0.007
Pb	0.00	2.08 (1.35, 3.55)	3.06 (1.31, 7.40)	2.34 (1.34, 4.87)	<0.001

Abbreviations: LOD, limit of detection. Data are presented as median (p25, p75); *p*-values were calculated by Mann–Whitney U test for the comparison of the exposed area and the reference area.

## Data Availability

De-identified participant data supporting this study are available upon reasonable request from the corresponding author.

## Supplementary Materials

The following supporting information can be downloaded at: https://www.mdpi.com/article/10.3390/toxics13070565/s1, Figure S1: Spearman correlations between metals; Figure S2: Adjusted OR (95%CI) for MetS according to quartiles of urinary metals in the multiple-metal models among subjects who resided more than 40 years in the study area; Figure S3: Weights representing the proportion of the positive or negative partial effect for each metal in the quantile g-computation model; Table S1: The quality control values and test results of the quality control urine samples; Table S2: Spike recovery results; Table S3: Association between urinary metals with MetS in the single-metal models in the all participants; Table S4: Association between urinary metals with MetS in the single-metal models in the exposed area; Table S5: Association between urinary metals with MetS in the single-metal models in the reference area.
