# Supplementary material for: Exposure to Metal Mixtures and Metabolic Syndrome in Residents Living near an Abandoned Lead–Zinc Mine: A Cross-Sectional Study
Source: Toxics. 2025 Jul 3;13(7):565. doi: 10.3390/toxics13070565 (PMC12300448; doi:10.3390/toxics13070565)
Supplement: Supplementary file 1 [file toxics-13-00565-s001.zip › toxics-3697276-supplementary.pdf]

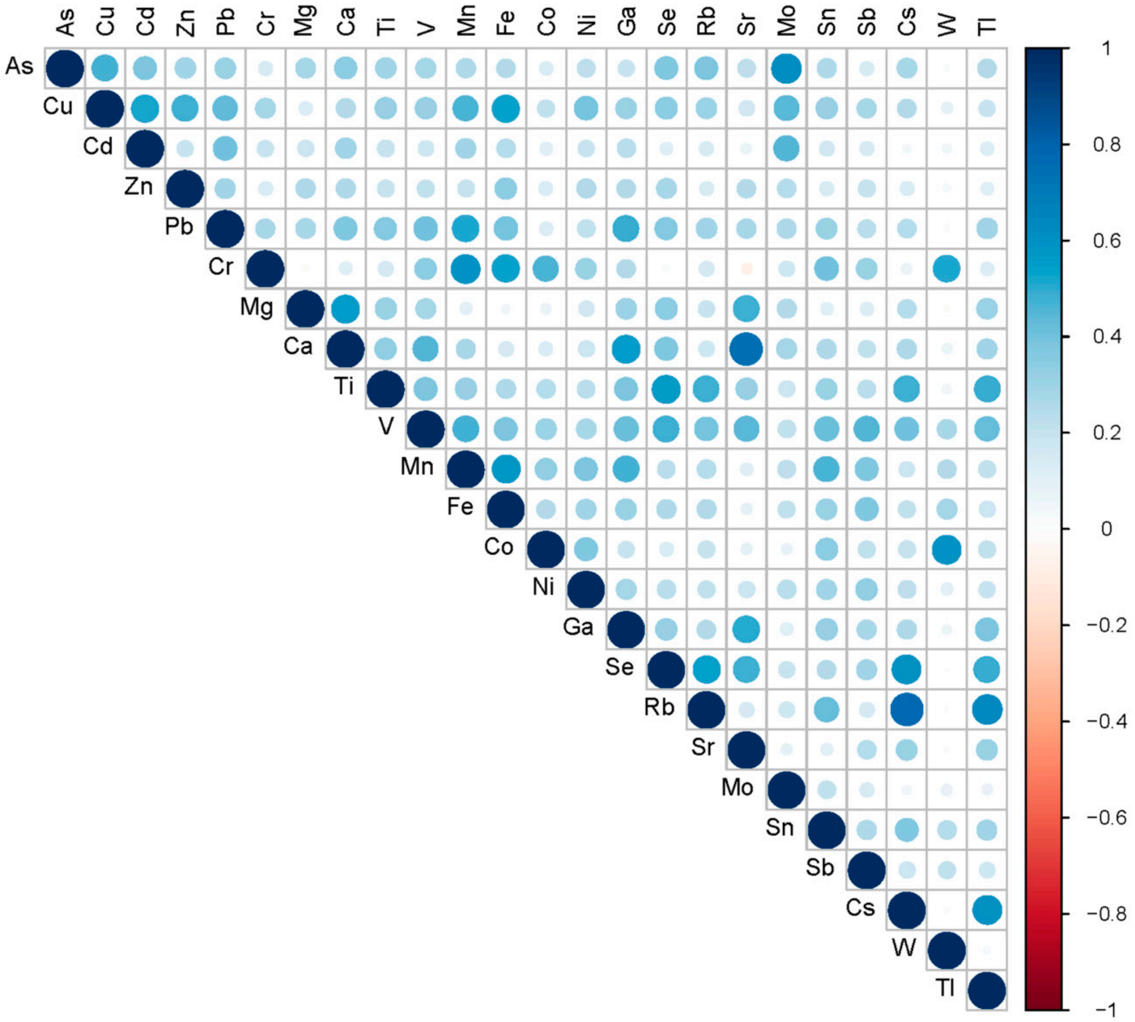

Figure S1. Spearman correlations between metals

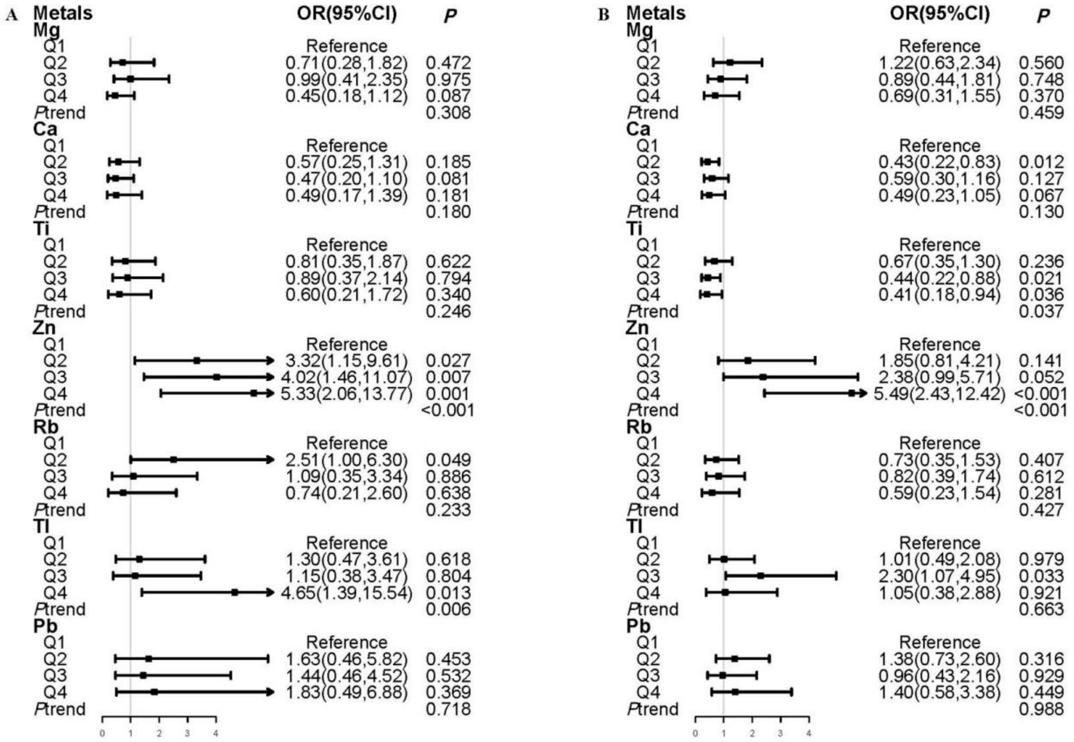

Figure S2. Adjusted OR (95%CI) for MetS according to quartiles of urinary metals in the multiple-metal models among subjects who resided more than 40 years in the study area (A) in exposed area, (B) in reference area. The metals selected by LASSO were simultaneously included in the logistic regression models, with adjustment for age, sex, ethnicity, education, smoking status, drinking status, physical activity, and BMI.

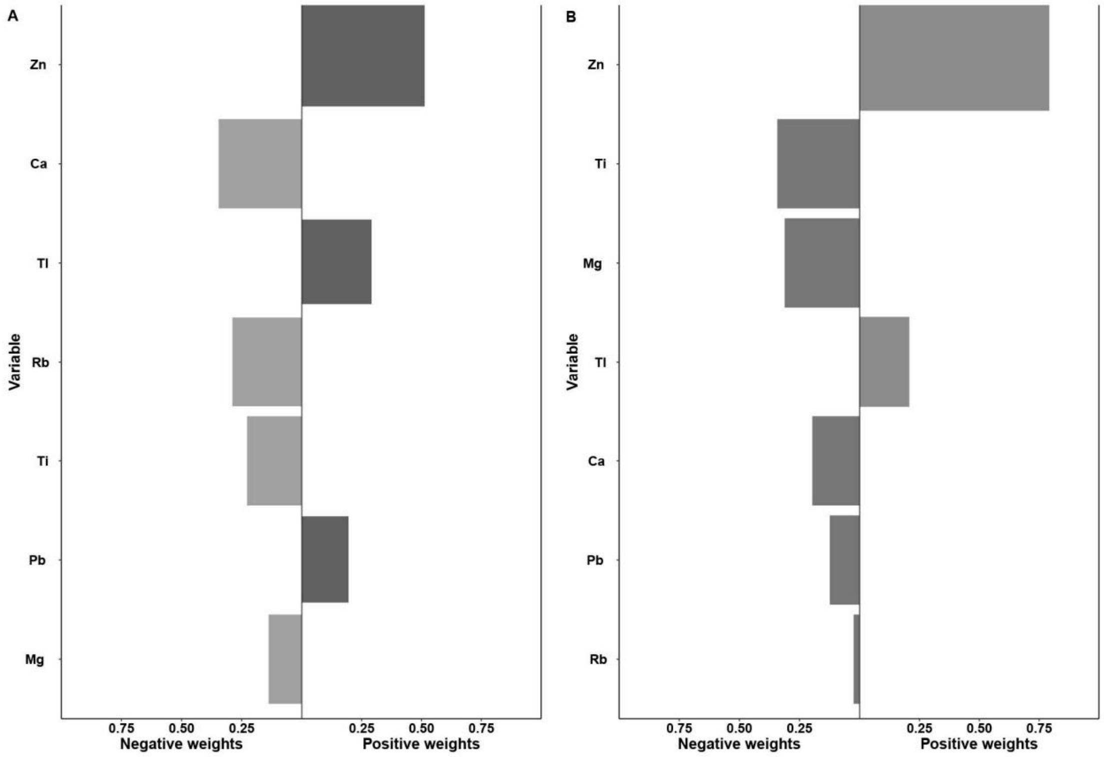

Figure S3. Weights representing the proportion of the positive or negative partial effect for each metal in the quantile g-computation model. (A) exposure area. (B) reference area.

Table S1

The quality control values and test results of the quality control urine samples

metal	Certified value (μg/L)	Observed value (μg/L)
Mg	81600-110800	108132.87
V	18.4-27.7	23.89
Cr	27.7-33.2	31.43
Mn	7.4-11.2	8.48
Fe	48.8-73.3	69.84
Co	10.2-15.3	12.68
Ni	30.6-46.1	32.67
Cu	62-74	70.55
Zn	136-205	193.61
As	173-223	199.94
Se	51.9-80.4	68.78
Cd	3.7-5.6	4.86
Sn	36.8-55.3	47.76
Sb	83-126	101.82
Tl	6.7-10	8.62
Pb	70.4-86.4	76.19

Table S2

Spike recovery results

Metals	Low concentrations		Medium concentrations		High concentrations	
	Spike Concentrations (μg/L)	Spike recovery rates (%)	Spike Concentrations (μg/L)	Spike recovery rates (%)	Spike Concentrations (μg/L)	Spike recovery rates (%)
Ca	3002	101.30	5255	101.25	8010	95.96
Ti	2	104.06	5	102.71	10	98.84
Ga	2	99.28	5	99.29	10	100.05
Rb	22	78.50	55	98.97	110	99.91
Sr	2	119.80	5	108.08	10	100.95
Mo	2	102.35	5	103.05	10	97.05
Cs	2	101.83	5	102.48	10	102.81
W	2	109.77	5	100.97	10	101.56

Table S3

Association between urinary metals with MetS in the single-metal models in the all participants (n=1744)

Metals (μg/g creatinine)	OR (95% CI)				*P*-trend
	Q1	Q2	Q3	Q4	
Mg(mg/g creatinine)	≤23.02	23.02 - 37.68	37.68 - 55.48	≥ 55.48	
	1.00	0.86(0.57,1.29)	0.77(0.51,1.16)	0.61(0.38,0.99)	0.037
Ca(mg/g creatinine)	≤51.53	51.53 - 85.81	85.81- 134.20	≥ 134.20	
	1.00	0.59(0.38,0.89)	0.58(0.38,0.88)	0.72(0.46,1.12)	0.072
Ti	≤16.27	16.27 - 22.50	22.50 - 30.05	≥ 30.05	
	1.00	0.88(0.59,1.33)	0.71(0.45,1.11)	0.63(0.39,1.02)	0.04
V	≤ 0.16	0.16 - 0.22	0.22 - 0.32	≥ 0.32	
	1.00	1.03(0.68,1.54)	0.58(0.37,0.92)	0.67(0.43,1.04)	0.021
Cr	≤ 0.19	0.19 - 0.34	0.34 - 0.74	≥ 0.74	
	1.00	0.64(0.41,0.99)	0.81(0.53,1.23)	0.72(0.45,1.13)	0.326
Mn	≤ 0.15	0.15 - 0.32	0.32 - 0.72	≥ 0.72	
	1.00	1.03(0.68,1.57)	0.66(0.41,1.05)	0.95(0.6,1.51)	0.531
Fe	≤ 9.23	9.23 - 14.36	14.36 - 25.59	≥ 25.59	
	1.00	1.78(1.13,2.8)	1.79(1.13,2.83)	1.56(0.97,2.51)	0.158
Co	≤ 0.16	0.16 - 0.27	0.27 - 0.55	≥ 0.55	
	1.00	1.83(1.2,2.81)	1.62(1.04,2.54)	1.12(0.71,1.77)	0.88
Ni	≤ 0.97	0.97 - 1.61	1.61 - 2.69	≥ 2.69	
	1.00	1.06(0.68,1.66)	1.13(0.72,1.76)	1.09(0.69,1.72)	0.667
Cu	≤ 9.77	9.77 - 12.63	12.63 - 17.52	≥ 17.52	

	1.00	1.18(0.72,1.91)	2.03(1.27,3.25)	2.04(1.29,3.22)	<0.001
Zn	≤ 188.94	188.94 - 272.35	272.35 - 388.68	≥ 388.68	
	1.00	1.73(1.01,2.96)	2.08(1.23,3.53)	3.62(2.21,5.95)	<0.001
Ga	≤ 0.14	0.14 - 0.25	0.25 - 0.48	≥ 0.48	
	1.00	0.76(0.49,1.16)	0.87(0.56,1.36)	0.85(0.55,1.33)	0.628
As	≤ 24.66	24.66 - 35.58	35.58 - 51.29	≥ 51.29	
	1.00	1.6(1.03,2.47)	1.19(0.76,1.88)	1.32(0.82,2.15)	0.400
Se	≤ 16.63	16.63 - 21.33	21.33 - 27.80	≥ 27.80	
	1.00	1.39(0.87,2.21)	1.65(1.02,2.67)	0.88(0.5,1.54)	0.842
Rb(mg/g creatinine)	≤ 1.24	1.24 - 1.71	1.71 - 2.42	≥ 2.42	
	1.00	1.20(0.77,1.87)	1.25(0.82,1.93)	0.80(0.48,1.35)	0.513
Sr	≤ 38.64	38.64 - 65.82	65.82 - 103.21	≥ 103.21	
	1.00	0.9(0.59,1.40)	0.96(0.6,1.53)	0.72(0.44,1.18)	0.248
Mo	≤ 48.79	48.79 - 76.66	76.66 - 126.29	≥ 126.29	
	1.00	1.20(0.78,1.86)	1.56(0.99,2.44)	1.12(0.68,1.86)	0.402
Cd	≤ 1.60	1.60 - 2.86	2.86 - 5.22	≥ 5.22	
	1.00	1.07(0.68,1.68)	1.32(0.82,2.13)	1.19(0.71,2.01)	0.401
Sn	≤ 0.37	0.37 - 0.53	0.53 - 0.84	≥ 0.84	
	1.00	0.71(0.47,1.07)	0.80(0.52,1.24)	0.52(0.32,0.85)	0.015
Sb	≤ 0.06	0.06 - 0.08	0.08 - 0.13	≥ 0.13	
	1.00	1.28(0.82,2.00)	1.21(0.77,1.89)	1.02(0.65,1.59)	0.965
Cs	≤ 6.21	6.21 - 8.21	8.21 - 10.85	≥ 10.85	
	1.00	0.99(0.64,1.52)	1.04(0.66,1.63)	0.68(0.4,1.15)	0.191
W	≤ 0.12	0.12 - 0.28	0.28 - 0.85	≥ 0.85	

	1.00	0.98(0.64,1.5)	1.08(0.71,1.64)	0.79(0.5,1.24)	0.335
Tl	≤ 0.31	0.31 - 0.46	0.46 - 0.69	≥ 0.69	
	1.00	0.97(0.63,1.5)	1.12(0.72,1.74)	1.08(0.66,1.77)	0.659
Pb	≤ 1.34	1.34 - 2.34	2.34 - 4.87	≥ 4.87	
	1.00	1.02(0.65,1.59)	0.95(0.62,1.47)	1.16(0.73,1.83)	0.551

Metals were individually included in the multivariable generalized linear regression models, with adjustment for age, sex, ethnicity, education, smoking status, drinking status, physical activity, BMI, and residence area as a random effect.

Table S4

Association between urinary metals with MetS in the single-metal models in the exposed area (n=723)

Metals	OR (95% CI)				*P*-trend
	Q1	Q2	Q3	Q4	
Mg	1.00	0.81(0.39,1.69)	1.21(0.65,2.25)	0.80(0.40,1.63)	0.773
Ca	1.00	0.68(0.34,1.38)	0.70(0.35,1.38)	1.01(0.48,2.10)	0.890
Ti	1.00	1.20(0.61,2.40)	1.20(0.59,2.42)	1.13(0.53,2.43)	0.722
V	1.00	2.03(1.01,4.10)	1.18(0.54,2.58)	1.52(0.67,3.44)	0.530
Cr	1.00	1.15(0.43,3.06)	1.69(0.66,4.29)	1.87(0.77,4.53)	0.093
Mn	1.00	2.00(0.69,5.79)	1.65(0.55,4.93)	2.71(0.94,7.82)	0.052
Fe	1.00	2.48(1.07,5.74)	3.94(1.69,9.18)	2.45(1.06,5.70)	0.071
Co	1.00	2.91(1.35,6.29)	2.39(1.09,5.26)	2.23(1.08,4.60)	0.177
Ni	1.00	2.04(1.00,4.16)	1.24(0.56,2.79)	2.07(1.00,4.29)	0.097
Cu	1.00	1.79(0.71,4.51)	4.87(2.03,11.72)	3.12(1.34,7.26)	0.004
Zn	1.00	3.85(1.55,9.56)	4.58(1.90,11.05)	5.59(2.46,12.69)	<0.001
Ga	1.00	1.25(0.58,2.70)	1.44(0.65,3.21)	1.54(0.72,3.30)	0.243
As	1.00	2.42(1.11,5.27)	2.18(0.97,4.93)	1.61(0.68,3.85)	0.346
Se	1.00	1.93(0.97,3.84)	3.07(1.57,5.98)	1.43(0.56,3.68)	0.036
Rb	1.00	2.59(1.20,5.59)	2.49(1.14,5.45)	1.46(0.59,3.63)	0.396
Sr	1.00	1.01(0.55,1.86)	1.22(0.59,2.51)	1.00(0.39,2.55)	0.778
Mo	1.00	2.07(0.84,5.11)	3.37(1.41,8.05)	2.02(0.80,5.09)	0.136
Cd	1.00	1.83(0.74,4.52)	1.23(0.52,2.91)	1.52(0.68,3.36)	0.536
Sn	1.00	2.21(0.89,5.49)	2.30(0.93,5.69)	1.36(0.52,3.56)	0.972
Sb	1.00	1.57(0.80,3.08)	2.10(1.06,4.19)	1.21(0.57,2.56)	0.407
Cs	1.00	1.81(0.91,3.62)	1.82(0.87,3.79)	1.54(0.66,3.57)	0.218

W	1.00	0.92(0.40,2.11)	1.14(0.57,2.30)	1.10(0.57,2.13)	0.672
Tl	1.00	1.53(0.73,3.21)	1.27(0.60,2.68)	2.68(1.23,5.84)	0.020
Pb	1.00	1.54(0.56,4.19)	1.75(0.83,3.69)	2.01(0.96,4.19)	0.076

Metals were individually included in the multivariable generalized linear regression models, with adjustment for age, sex, ethnicity, education, smoking status, drinking status, physical activity, and BMI.

Table S5

Association between urinary metals with MetS in the single-metal models in the reference area (n=1021)

Metals	OR (95% CI)				*P*-trend
	Q1	Q2	Q3	Q4	
Mg	1.00	0.82(0.5,1.36)	0.57(0.33,0.98)	0.47(0.25,0.92)	0.010
Ca	1.00	0.50(0.30,0.86)	0.49(0.29,0.83)	0.54(0.31,0.96)	0.015
Ti	1.00	0.78(0.47,1.31)	0.51(0.29,0.91)	0.47(0.25,0.87)	0.007
V	1.00	0.69(0.40,1.18)	0.38(0.22,0.68)	0.43(0.25,0.74)	0.001
Cr	1.00	0.61(0.36,1.02)	0.72(0.43,1.21)	0.44(0.21,0.94)	0.038
Mn	1.00	0.92(0.57,1.48)	0.54(0.30,0.96)	0.59(0.31,1.15)	0.041
Fe	1.00	1.51(0.88,2.62)	1.21(0.69,2.14)	1.36(0.74,2.50)	0.523
Co	1.00	1.49(0.88,2.52)	1.30(0.75,2.27)	0.71(0.38,1.32)	0.190
Ni	1.00	0.73(0.41,1.29)	1.08(0.62,1.88)	0.74(0.41,1.33)	0.527
Cu	1.00	0.99(0.55,1.76)	1.41(0.79,2.51)	2.12(1.17,3.82)	0.007
Zn	1.00	1.13(0.59,2.15)	1.31(0.68,2.55)	3.09(1.66,5.76)	<0.001
Ga	1.00	0.59(0.35,1.01)	0.71(0.40,1.25)	0.64(0.35,1.16)	0.204
As	1.00	1.33(0.78,2.27)	0.78(0.43,1.41)	1.33(0.72,2.45)	0.704
Se	1.00	0.80(0.42,1.54)	0.76(0.39,1.50)	0.48(0.24,0.98)	0.031
Rb	1.00	0.79(0.44,1.40)	0.90(0.52,1.55)	0.63(0.33,1.22)	0.236
Sr	1.00	0.65(0.35,1.21)	0.61(0.33,1.11)	0.47(0.25,0.87)	0.022
Mo	1.00	1.04(0.63,1.72)	1.09(0.62,1.92)	0.90(0.45,1.80)	0.898
Cd	1.00	0.94(0.54,1.63)	1.61(0.87,2.98)	1.05(0.45,2.46)	0.435
Sn	1.00	0.49(0.30,0.81)	0.63(0.36,1.11)	0.42(0.22,0.78)	0.010
Sb	1.00	1.05(0.57,1.93)	0.87(0.49,1.55)	0.85(0.48,1.52)	0.487
Cs	1.00	0.60(0.34,1.06)	0.69(0.38,1.25)	0.40(0.20,0.79)	0.015

W	1.00	1.01(0.61,1.67)	1.03(0.60,1.76)	0.56(0.28,1.11)	0.113
Tl	1.00	0.70(0.41,1.20)	0.96(0.55,1.68)	0.59(0.30,1.14)	0.213
Pb	1.00	0.90(0.55,1.47)	0.74(0.42,1.30)	0.81(0.39,1.66)	0.422

Metals were individually included in the multivariable generalized linear regression models, with adjustment for age, sex, ethnicity, education, smoking status, drinking status, physical activity, and BMI.
